# Skiascopy and Its Practical Application to the Study of Refraction

**Published:** 1897-09

**Authors:** 


**Skiascopy and its Practical Application to the Study of Refraction.** By EDWARD JACKSON, M.D. Second Edition. Pp. 108. Philadelphia: The Edwards & Docker Co. 1896.

The first edition of this book supplied a want much felt, since even the best text-books of ophthalmology give very scanty information on this subject. The various normal and abnormal phenomena of the shadow test are on the whole very clearly described and explained by Dr. Jackson. Every difficulty is dealt with, and every application of the test considered. With the second edition to refer to, no student of ophthalmology should have any great difficulty in thoroughly mastering the subject. We are glad to see that careful attention is directed to the information to be gained from observation of the fundus reflex at different distances, and also to the size and position of the source of light—important matters which are far too frequently neglected. Some of the paragraphs are not so intelligible as they might be; for instance, those on the "Position of Observer for Greatest Accuracy." If the book has a fault, it is that it is laboriously minute in dealing with the possibilities of skiascopy, some of which have scarcely any practical value, and it seems hardly necessary to devote separate chapters to the use of the concave and plane mirror respectively. The diagrams, illustrations, and letterpress are excellent, and the substitution of graphic or geometric explanations for mathematical formulæ will meet with almost universal approval.

